# Use of different RT-QuIC substrates for detecting CWD prions in the brain of Norwegian cervids

**DOI:** 10.1038/s41598-019-55078-x

**Published:** 2019-12-09

**Authors:** Edoardo Bistaffa, Tram Thu Vuong, Federico Angelo Cazzaniga, Linh Tran, Giulia Salzano, Giuseppe Legname, Giorgio Giaccone, Sylvie L. Benestad, Fabio Moda

**Affiliations:** 1 Fondazione IRCCS Istituto Neurologico Carlo Besta, Division of Neurology 5 and Neuropathology, Milano, Italy; 2 Norwegian Veterinary Institute, Oslo, Norway; 3 Scuola Internazionale Superiore di Studi Avanzati (SISSA), Laboratory of Prion Biology, Department of Neuroscience, Trieste, Italy

**Keywords:** Biological techniques, Neuroscience

## Abstract

Chronic wasting disease (CWD) is a highly contagious prion disease affecting captive and free-ranging cervid populations. CWD has been detected in United States, Canada, South Korea and, most recently, in Europe (Norway, Finland and Sweden). Animals with CWD release infectious prions in the environment through saliva, urine and feces sustaining disease spreading between cervids but also potentially to other non-cervids ruminants (e.g. sheep, goats and cattle). In the light of these considerations and due to CWD unknown zoonotic potential, it is of utmost importance to follow specific surveillance programs useful to minimize disease spreading and transmission. The European community has already in place specific surveillance measures, but the traditional diagnostic tests performed on nervous or lymphoid tissues lack sensitivity. We have optimized a Real-Time Quaking-Induced Conversion (RT-QuIC) assay for detecting CWD prions with high sensitivity and specificity to try to overcome this problem. In this work, we show that bank vole prion protein (PrP) is an excellent substrate for RT-QuIC reactions, enabling the detection of trace-amounts of CWD prions, regardless of prion strain and cervid species. Beside supporting the traditional diagnostic tests, this technology could be exploited for detecting prions in peripheral tissues from live animals, possibly even at preclinical stages of the disease.

## Introduction

Chronic Wasting Disease (CWD) is a prion disease, a neurodegenerative disorder that affects cervid populations. It was first identified in 1967 in a captive mule deer from Colorado. CWD was then diagnosed across a wide area of North America^1^ where it is spreading extensively. It has also been reported in South Korea, as a consequence of infected animals imported from Canada^2^ and has most recently been reported in Europe^3^ (Norway, Finland and Sweden^4,5^). To date, CWD has been identified in mule deer (*Odocoileus hemionus*)^6^, black-tailed deer (*Odocoileus hemionus*), white-tailed deer (*Odocoileus virginianus*)^7^, elk (*Cervus canadensis*)^6,8^ moose (*Alces alces*)^9^, reindeer (*Rangifer tarandus tarandus*) and red deer (*Cervus elaphus*)^2,10–12^.

As in other prion diseases, the causative agent is considered to be an abnormally folded isoform of the prion protein (PrP^C^), named prion or PrP^Sc^^13^, which accumulates mostly in the central nervous system (CNS). PrP^C^ plays important roles in several physiological processes and is evolutionarily conserved amongst different mammalian species^3,6,14–19^. Misfolded PrP^Sc^ propagates through conformational templating where PrP^C^ is converted into PrP^Sc^ thus acquiring infectious features and sustaining disease^13,20^.

Horizontal transmission of CWD is highly efficient, through both animal-to-animal contact and exposure to environments contaminated with prion infected material (e.g. excreta, placenta or carcasses)^21–24^. Polymorphisms in the PrP gene (*Prnp*) are known to affect (i) animal susceptibility to CWD, (ii) transmission efficiency between species, (iii) clinical and (iv) neuropathological features of the disease^25–28^. For instance, deer PrP^C^ is characterized by having a glycine (G) or serine (S) at codon 96 of *Prnp*^29,30^; Moose PrP^C^ contains either lysine (K) or glutamine (Q) at position 109^31^; Elk PrP^C^ has either methionine (M) or leucine (L) at position 132^26^; Reindeer PrP^C^ has aspartic acid (D) or asparagine (N) at residue 176^31^; Mule deer PrP^C^ contains either serine (S) or phenylalanine (F) at position 225^32^ while Whitetail deer is characterized by having alanine (A) or glycine (G) at position 116 and glutamine (Q) or lysine (K) at position 226^28^. These amino acids determine important variability in disease phenotypes or susceptibility to CWD infection. For example, the expression of L at position 132 of elk PrP appears to confer, at least partially, resistance to CWD when compared to the more prevalent 132 M allele^33^. Similarly, the expression of S at position 96 of deer PrP is linked to reduced incidence of CWD^34^.

Thus, other than dictating CWD susceptibility, PrP polymorphisms seem to play an important role in prion strains selection^35–37^. Strains are extremely relevant in prion diseases since they determine variability in clinical phenotype of the disease, and influence the characteristics of PrP^Sc^ that acquires distinct biochemical properties (e.g., electrophoretic mobility in Western blot (WB) and analysis of glycoforms ratio after treatment with proteinase K) and differentially accumulates in specific brain areas^38–43^. Differences in strain behavior and features are known to rely on different abnormal conformations that could be acquired by PrP^Sc^. Strains conformational mutations can be further promoted during CWD transmission between species where PrP^Sc^ is subjected to important processes of selection and adaptation in the new host^36,44–46^.

Definite CWD diagnosis relies on *post-mortem* detection of PrP^Sc^ in the brainstem and the head lymph nodes^47–49^ using rapid tests (enzyme-linked immunosorbent assays (ELISA), WB and/or immunohistochemistry (IHC))^50–52^. PrP^Sc^ can be detected in peripheral lymphoid tissues (especially those associated with the alimentary canal) of most CWD affected animals, like in sheep affected with classical scrapie, before it can be detected in the brain^48,53,54^. For instance, IHC analyses of the palatine tonsils and recto-anal mucosa-associated lymphoid tissues (RAMALT) are used for an *ante-mortem* diagnosis of CWD in deer^49,55–57^. Nevertheless, in some CWD affected elks, while PrP^Sc^ is detected in the brain, the presence of PrP^Sc^ in the lymphoid tissues can be minimal or undetectable, which represents an important limitation for the *ante-mortem* diagnosis^58,59^. Similarly, Norwegian moose and red deer show PrP^Sc^ in the brain but PrP^Sc^ was not detected in lymphoid tissues^3,9,60^. Reindeer in Norway, conversely, have detectable lymphoid PrP^Sc^ accumulation even in animals where no PrP^Sc^ is detected in the CNS^3^.

However, at early stages of the disease, also the amount of PrP^Sc^ in peripheral lymph nodes is lower than the detection threshold of traditional diagnostic techniques^61^. In this case, the most reliable way to detect low titer of infectious prions is therefore by animal bioassay. Even though these experiments remain the gold standard for measuring low amounts of infectivity, they are time consuming, expensive and can therefore not be used as screening tests^62–65^.

Thanks to the recent development of *in vitro* cell-free amplification techniques, including the Protein Misfolding Cyclic Amplification (PMCA) and the Real Time Quaking Induced Conversion (RT-QuIC) assays, it is possible to detect trace-amounts of CWD prions in different peripheral tissues and biological fluids^66^. In particular, PMCA showed the presence of PrP^Sc^ in muscles^67^, feces^68,69^, saliva^70^, cerebrospinal fluid (CSF)^71^ and even the blood of animals at different stages of CWD infection^72,73^. Unfortunately, there is no universal PMCA substrate for amplifying CWD from different species. This technique, therefore would seem not to be the method of choice when analyzing samples that are collected in the field (feces or saliva) from species with unknown origin. In RT-QuIC, through alternate cycles of incubation and shaking, prions force the substrate of reaction (recombinant PrP) to adopt β-sheet structures^74^. These abnormally folded PrP proteins aggregate and form amyloid fibrils whose growth is monitored with Thioflavin-T (ThT) fluorescent dye. The assay has been used extensively for detecting CWD prions in nasal swabs^75^, lymphoid tissues^76^, CSF^71^, blood^77^, saliva^78,79^, urine^80^ and feces^81,82^ of animals at clinical and, sometimes, preclinical stages of disease. The presence of CWD prions in the saliva, urine and feces of affected animals suggests that substantial environmental contamination can occur during the entire course of the disease, especially considering that sialorrhea and polyuria are common in diseased animals^83–87^. Moreover, prions can persist in the environment for many years and thus represent a serious risk of future contamination upon restocking of CWD-exposed areas^1,88–93^.

For this reason, RT-QuIC due to its high sensitivity and high-throughput potential can have useful applications for *ante-mortem* identification of CWD infected animals. However, the technique detects different prion strains or strains belonging to different species with variable efficiency. To date, most of the RT-QuIC experiments described in the literature have been performed using primarily Syrian hamster or deer PrP as substrate of reaction (see Table 1). In the present study, RT-QuIC analyses were performed on serial dilutions of brain homogenates collected from both healthy and CWD-affected Norwegian moose, reindeer and red deer, using different substrates. We evaluated the ability of truncated PrP from Syrian hamster, bank vole, deer, reindeer and elk to detect brain derived PrP^Sc^ regardless of animal species or prion strain. This is also the first time where RT-QuIC has been used to analyze samples from CWD affected Norwegian reindeer.

**Table 1:** Summary of RT-QuIC substrates (PrP) used to analyze different tissues of CWD affected cervid species.

Species	Prion	Tissue analyzed	Substrate	Year	References
Deer	CWD	Brain	Deer PrP (24–234)	2010	^130^
Deer	CWD	Brain, urine and feces	Deer PrP (24–234)	2013	^80^
Deer	CWD	Saliva	Syrian hamster PrP (90–231)	2013	^79^
Deer	CWD	Blood	Syrian hamster PrP (90–231)	2013	^77^
Deer	CWD	CSF	Syrian hamster PrP (90–231)	2013	^71^
Deer	CWD	Retropharyngeal lymph node	Syrian hamster PrP (90–231)	2014	^131^
Deer	CWD	Brain, saliva	Syrian hamster PrP (90–231)	2015	^132^
Deer Elk	CWD	Brain	Bank vole (23–230)	2015	^100^
Deer	CWD	Saliva, urine	Syrian hamster PrP (90–231)	2015	^78^
Deer	CWD	Brain	Deer PrP (23–231)	2015	^124^
Deer	CWD	RAMALT, nasal brush	Syrian hamster PrP (90–231)	2016	^75^
Elk	CWD	RAMALT, nasal brush	Syrian hamster PrP (90–231)	2016	^76^
Deer	CWD	Feces	Syrian hamster PrP (90–231)	2016	^69^
Deer	CWD	Brain, lymphoid tissues	Syrian hamster PrP (90–231)	2017	^133^
Deer	CWD	Ovary tissue, uterine tissue, placentome, amniotic and allantoic fluids	Syrian hamster PrP (90–231)	2017	^134^
Deer	CWD	Brain	Deer PrP (24–234)	2017	^135^
Deer	CWD	Gastrointestinal tissues (e.g. omasum, abomasum, colon, cecum) and lymphoid tissues (e.g. spleen, tonsils)	Syrian hamster PrP (90–231)	2017	^136^
Elk	CWD	Feces	Syrian hamster PrP (90–231)	2017	^82^
Elk	CWD	RAMALT	Syrian hamster PrP (90–231)	2017	^137^
Deer	CWD	Saliva	Syrian hamster PrP (90–231)	2017	^138^
Deer Elk	CWD	RAMALT	White-tailed deer PrP (25–232)	2017	^139^
			Mule deer PrP (25–232)		
			Fallow deer PrP (25–232)		
			Elk PrP (25–232)		
			Reindeer PrP (25–232)		
Elk	CWD	Blood, rectal biopsy	Syrian hamster PrP (90–231)	2018	^140^
Deer Elk	CWD	Retropharyngeal lymph node, brain	Syrian hamster PrP (90–231)	2018	^60^
Deer	CWD	Saliva	Syrian hamster PrP (90–231)	2018	^70^
Elk	CWD	Brain	Elk PrP (23–231)	2018	^141^
Elk	CWD	Brain	Syrian hamster PrP (90–231)	2019	^4^
Deer	CWD	Brain	Syrian hamster PrP (90–231)	2019	^142^
Deer	CWD	Brain, eyelids	Syrian hamster PrP (90–231)	2019	^143^

Our results indicate that the use of bank vole PrP provided efficient prion detection in all CWD affected animals, even in the instances where traditional diagnostic methods (WB, IHC or ELISA) failed to demonstrate the presence of PrP^Sc^. This optimized RT-QuIC assay could therefore be used as a screening test for CWD detection. It could also be a useful research tool for analyzing other tissues with low levels of PrP^Sc^ like peripheral tissues from live animals or excreta (such as urine, saliva and blood) that can be collected through non-invasive procedures. Moreover, although there are no documented cases of natural interspecies transmission of CWD to non-cervid animals, many livestock (especially sheep and goats) which share their habitat with diseased cervids will be exposed to prions and could potentially be infected. Although there is a so-called “species barrier”, which is related to differences in PrP sequences between donor and acceptor animals that can limit the efficiency of prion transmission between species (spillover phenomenon that occur for instance from cervids to small ruminants), the potential risk of breaking such barrier cannot be excluded. For instance, CWD transmission to other species (especially human) cannot be completely ruled out at present^64,94,95^. For this reason, identifying preclinical CWD affected animals is of fundamental importance for minimizing horizontal disease transmission.

## Results

### TeSeE^TM^ WB analysis detects prions in brain or lymph nodes of CWD affected animals

**Figure 1:**
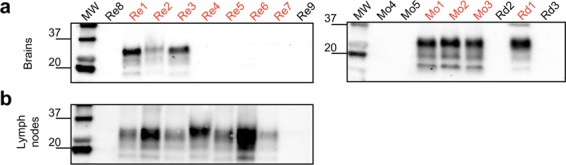
TeSeE^TM^ WB results of brain and lymph nodes collected from the healthy (black) and CWD affected (red) animals included in the study. (**a**) PrP^res^ was detected in all brain homogenates of CWD affected moose (Mo1, Mo2, Mo3), red deer (Rd1) and 3 brain homogenates (out of 7) of CWD affected reindeer. Notably, no PrP^res^ was detected in the brains of CWD affected reindeer 4, 5, 6, and 7; (**b**) PrP^res^ was however detected in the lymph nodes of all the CWD affected reindeer. PrP^res^ was not found in brain and lymph nodes of healthy animals. Numbers on the left of the Western blots indicate molecular weights (kDa).

The CWD affected animals used in this study were identified through the Norwegian surveillance program for CWD that has been started in 2016. These animals were firstly diagnosed by TeSeE^TM^ ELISA test and then confirmed by WB analysis (diagnostic statuses are summarized in Table 2). To verify the presence of different distribution patterns of CWD prions in both brain and lymph nodes WB analyses were finally performed. Proteinase K resistant PrP (PrP^res^) was found in the samples from all the CWD affected moose (Mo1, Mo2 and Mo3) and red deer (Rd1), while PrP^res^ was detected only in 3 out of 7 brain samples of CWD affected reindeer (Fig. 1a). In contrast, PrP^res^ was not found in the lymph nodes of CWD affected moose and red deer but always detected in lymph nodes of the CWD affected reindeer (Re1-Re7) (Fig. 1b). PrP^res^ was not detected in any of the samples (brain or lymph node) collected from healthy animals.

**Table 2:** Demographic information and TeSeE^TM^ WB results of the Norwegian animals included in this study.

Species	Sample ID	Status	Geographic origin	Sex	Age(y)	PrP^res^ detection	
						Brain	LN
*Alces alces*	Mo1	CWD	Lierne	Female	13	+	−
	Mo2	CWD	Selbu	Female	13	+	−
	Mo3	CWD	Selbu	Female	14	+	−
	Mo4	Healthy	Råde	*Unknown*	>1	−	−
	Mo5	Healthy	Voss	Male	>1	−	−
*Cervus elaphus*	Rd1	CWD	Gjemnes	Female	16	+	−
	Rd2	Healthy	Eid	Female	>1	−	−
	Rd3	Healthy	Årdal	Male	>1	−	−
*Rangifer tarandus tarandus*	Re1	CWD	Nordfjella	Male	>1	+	+
	Re2	CWD	Nordfjella	Female	>1	+	+
	Re3	CWD	Nordfjella	Male	>1	+	+
	Re4	CWD	Nordfjella	Female	>1	−	+
	Re5	CWD	Nordfjella	Male	1.5	−	+
	Re6	CWD	Nordfjella	Female	>1	−	+
	Re7	CWD	Nordfjella	Male	8	−	+
	Re8	Healthy	Lom	Male	>1	−	−
	Re9	Healthy	Lom	Female	>1	−	−

### RT-QuIC analysis with bank vole PrP enables efficient prion detection in brain samples from cervids where PrP^Sc^ was biochemically detected

**Figure 2:**
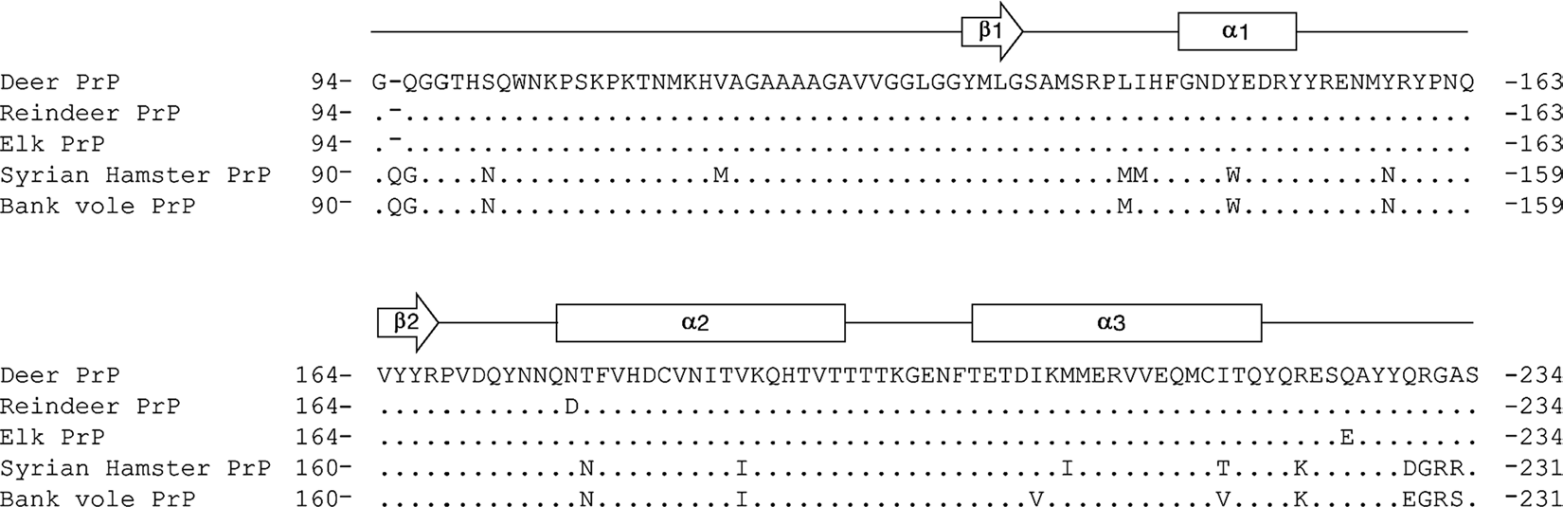
Amino acid sequences of recombinant PrP proteins used for RT-QuIC experiments. The amino acid sequence of deer PrP was used as reference for aligning the sequences of reindeer, elk, Syrian hamster and bank vole PrP. Arrows and rectangles indicate beta-sheets (β1 and β2) and alpha helix (α1, α2 and α3) secondary structures, respectively.

With the aim of evaluating the efficiency of different substrates in detecting CWD prions in different cervid species, RT-QuIC experiments were performed using recombinant PrP proteins with amino acidic sequences belonging to the following animal species: Syrian hamster, bank vole (109 M), deer (96 G), reindeer (176D) and elk (132 M) (Fig. 2). According to the most recent publications (see Table 1), we decided to use truncated proteins since the C-terminal protein domain allows prion detection with high sensitivity and specificity^96,97^. Evaluation of the overall RT-QuIC performance was based on brain samples collected from CWD affected animals where PrP^Sc^ was detected by means of TeSeE^TM^ ELISA and WB. Brain homogenates of Rd1, Mo1, Mo2, Mo3, Re1, Re2 and Re3 were diluted from 10^−5^ to 10^−7^ and subjected to RT-QuIC analysis. Brain samples of healthy animals (Rd2, Rd3, Mo4, Mo5, Re8 and Re9) were used as controls.

**Figure 3:**
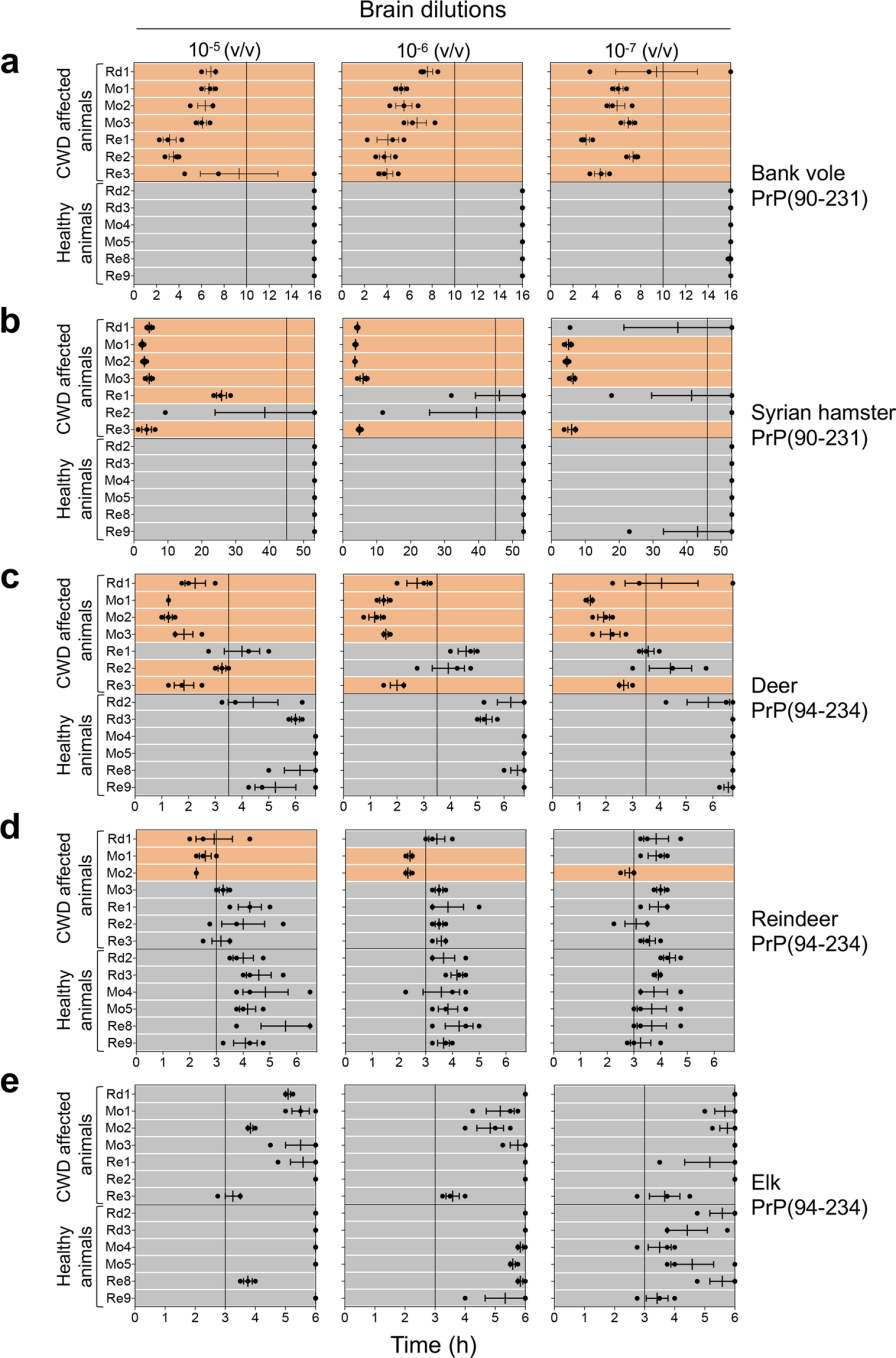
RT-QuIC results of CWD affected animals with detectable PrP^Sc^ in the brain. RT-QuIC analysis of serial brain homogenate dilutions (from 10^−5^ to 10^−7^) from CWD affected animals and controls with (**a**) bank vole, (**b**) Syrian hamster, (**c**) deer, (**d**) reindeer and (**e**) elk recombinant truncated PrP. Each sample was analyzed in triplicate and black dots indicate the time taken for each replicate to reach the fluorescence threshold (lag phase). The vertical line indicates the time threshold set up for each PrP substrate. Rd: red deer, Mo: moose; Re: reindeer. Mean value and standard error of the mean (S.E.M) are shown.

Regardless of the animal species or brain dilution, bank vole PrP enabled PrP^Sc^ detection with a higher sensitivity and specificity compared to the other tested substrates (Fig. 3a). Particularly, all CWD affected animals’ samples induced RT-QuIC seeding activity within 10 hours while those of healthy controls did not. The reaction was stopped at 16 hours because at this point negative controls started to induce unspecific seeding activity.

Compared to the bank vole PrP, Syrian hamster PrP substrate did not detect all CWD affected animals’ samples and the sensitivity decreased at higher dilutions. In particular, one (Re2), two (Re1 and Re2) or three (Re1, Re2 and Rd1) CWD affected animals’ samples were not detected at 10^−5^, 10^−6^ or 10^−7^ dilutions, respectively (Fig. 3b). Notably, this substrate hardly detected Norwegian reindeer CWD prions compared to the moose and red deer ones. The reaction was stopped at 53 hours and none of the negative controls induced unspecific reaction. We analyzed whether the prion protein sequence homology between CWD prions and substrate could increase the power of discrimination between prion affected and healthy animals. We then analyzed the samples using PrP substrates with amino acid sequences of deer, reindeer and elk. Surprisingly, while deer PrP (Fig. 3c) was still able to detect CWD affected animals’ samples with sensitivity and specificity quite comparable to that of Syrian hamster PrP, reindeer (Fig. 3d) and elk PrP (Fig. 3e) were characterized by a very rapid aggregation in both the positive and the negative samples. Even with a time threshold of 3 hours, we could not clearly discriminate between CWD affected animals and healthy controls. Thus, PrP substrates with cervid sequences appear to be less efficient in detecting CWD prions than those with bank vole and Syrian hamster sequences.

**Figure 4:**
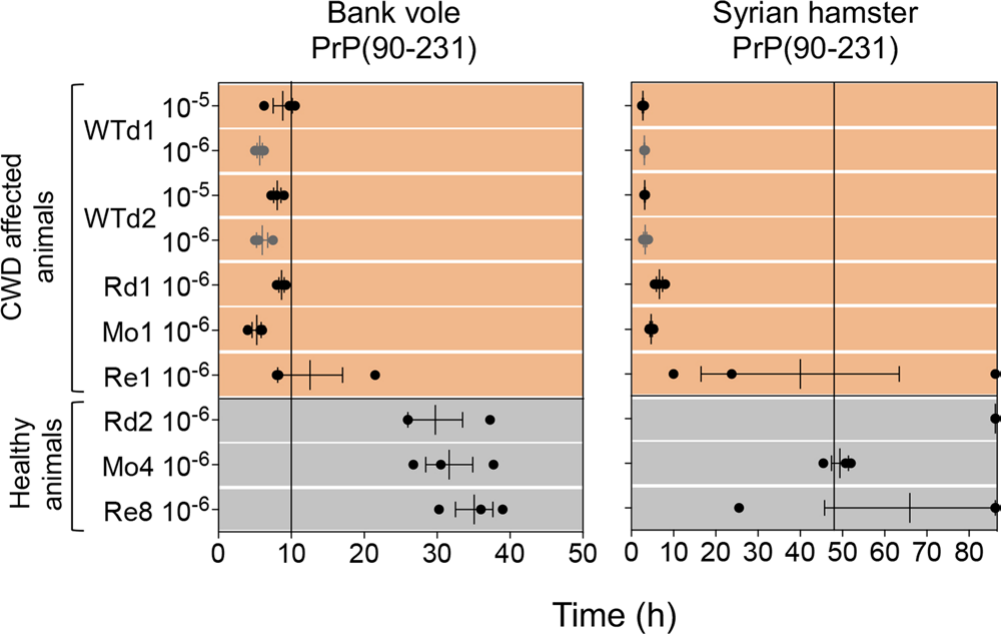
RT-QuIC results of CWD affected Norwegian and North American cervid species. Brain homogenates were diluted at 10^−5^ and/or 10^−6^ and analyzed by RT-QuIC using bank vole and Syrian hamster recombinant truncated PrP. Each sample was analyzed in triplicate and black dots indicate the time taken for each replicate to reach the fluorescence threshold (lag phase). The vertical line indicates the time threshold set up for each PrP substrate. WTd: white tailed deer (North America); Rd: red deer; Mo: moose; Re: reindeer. Mean value and standard error of the mean (S.E.M) are shown.

Two brain samples from CWD affected white-tailed deer (WTd) from North America were included in the RT-QuIC analysis to verify whether the overall performance of the assay could have been influenced by the origin of the CWD prions (Norway *vs* North America). The two isolates were provided to the Norwegian Veterinary Institute in Oslo in 2006 as part of a ring trial (courtesy of Aru Balachandran, Canadian Food Inspection Agency, Alberta, Canada). In this case, their seeding activities were similar to that of Norwegian CWD affected deer, in terms of lag phase and fluorescence intensity (Fig. 4 and see Supplementary Fig. S1).

### RT-QuIC analysis with bank vole PrP enables efficient prion detection in reindeer brain samples where PrP^Sc^ was not biochemically detected

**Figure 5:**
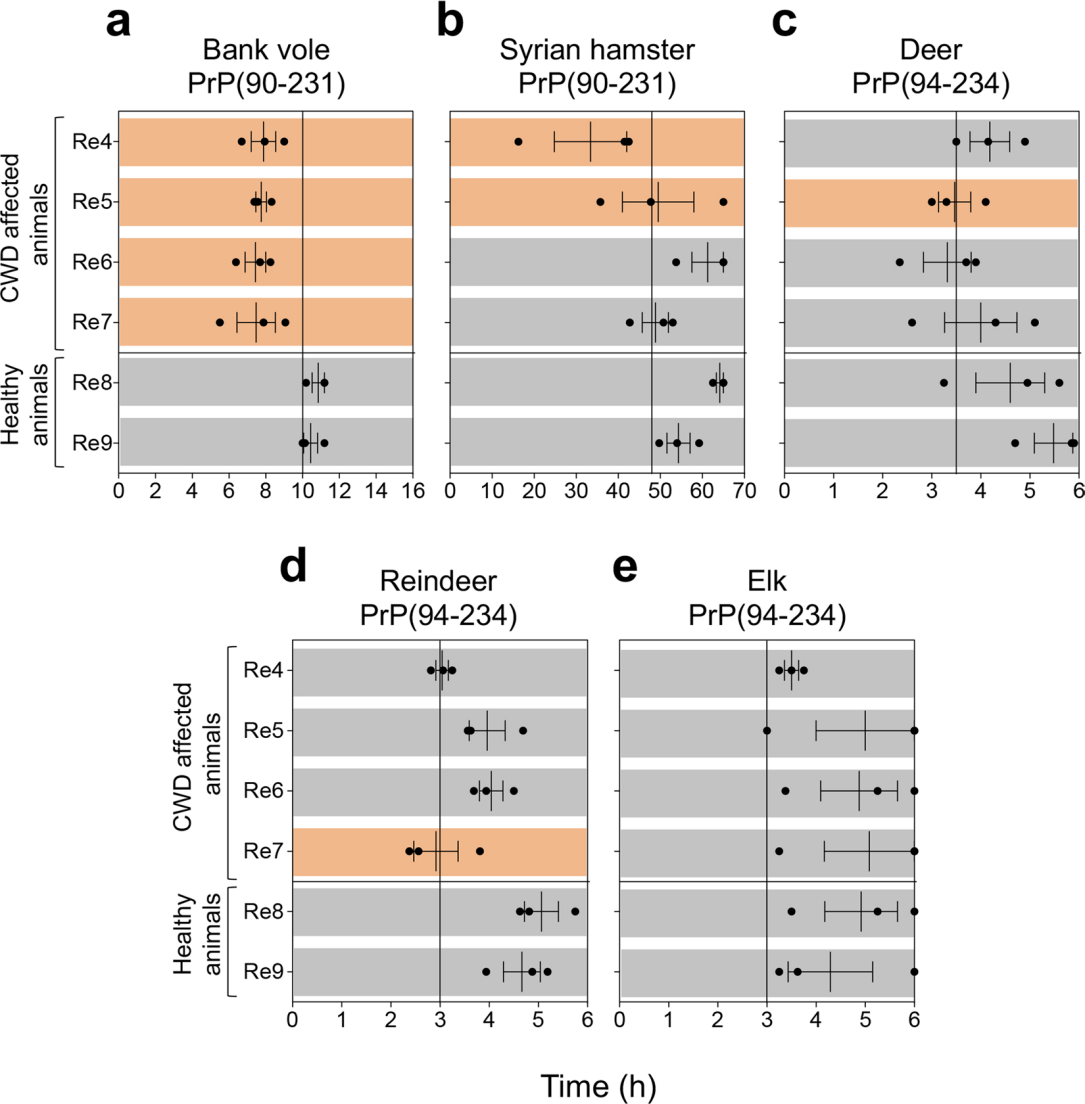
RT-QuIC results of CWD affected reindeer where PrP^Sc^ was not biochemically detected in the brain. Brain homogenates were diluted at 10^−5^ and analyzed by RT-QuIC using (**a**) bank vole, (**b**) Syrian hamster, (**c**) deer, (**d**) reindeer and (**e**) elk recombinant truncated PrP. Each sample was analyzed in triplicate and black dots indicate the time to reach the fluorescence threshold (lag-phase) of each replicate. The vertical line indicates the time threshold set up for each PrP substrate. Rd: red deer; Mo: moose; Re: reindeer. Mean value and standard error of the mean (S.E.M) are shown.

Finally, we have analyzed samples collected from CWD affected reindeer (Re4, Re5, Re6, Re7) where PrP^Sc^ had only been detected in the lymph nodes and not the brain with TeSeE^TM^ ELISA and WB. We decided not to perform serial dilutions given the lack of WB signal in the brain samples and chose a dilution of 10^−5^. As previously observed, the bank vole PrP was the most efficient at detecting prions in all the CWD affected reindeer (Fig. 5a). The Syrian hamster PrP did not detect 2 out of 4 CWD samples (Re6 and Re7) (Fig. 5b); whilst deer (Fig. 5c) and reindeer PrP (Fig. 5d) did not detect 3 out of the 4 CWD samples (Re4, Re6, Re7 and Re4, Re5, Re6, respectively). Elk PrP did not detect any of them (Fig. 5e).

## Discussion

First discovered in deer in Colorado many decades ago, CWD rapidly spread to many other American states^98^ and Canada^10^. In April 2016, the disease was diagnosed in a Norwegian reindeer from the Nordfjella area^3^. This was the first case of CWD in Europe, and the first reindeer reported with naturally occurring CWD. This disease is contagious within cervid populations and can efficiently transmit directly between animals or through the environment. Healthy animals can be infected after close contact with saliva, urine and feces of affected ones (direct horizontal transmission) or after being exposed to environments contaminated with excreta or carcasses of diseased animals (indirect horizontal transmission). Prions persist in the environment for long time and contribute significantly to disease spreading.

In Europe surveillance programs are aimed at detecting the presence of CWD in wild and farmed cervids. The validated and approved diagnostic tests require animals to be sacrificed for sampling the CNS (e.g. brainstem) and/or lymphoid tissue for ELISA, WB and IHC based analyses^61^. Although these tests reach high levels of diagnostic accuracy for CWD, PrP^Sc^ accumulation in brain and lymphoid tissues can be lower than the detection threshold of the tests, especially in the early stages of disease. In addition, different CWD prion strains can affect test performance, as in the case of Nor98/atypical scrapie^99^.

In this work we evaluated the efficiency of the highly sensitive RT-QuIC assay in detecting low amounts of CWD prion in different Norwegian cervid species. Our aim was to set up optimal conditions for PrP^Sc^ detection, regardless of prion strains and animal species. For this reason, brain homogenates of CWD affected moose, red deer and reindeer were serially diluted and subjected to RT-QuIC analysis performed using Syrian hamster, bank vole, deer, reindeer and elk PrP as reaction’s substrates.

Our results indicated that the bank vole PrP enabled CWD prion detection in every brain dilution of all cervid species, especially in reindeer where PrP^Sc^ detection was more challenging compared to the other species and we could clearly discriminate CWD affected animals from healthy controls. A slightly less efficient detection of CWD prions was observed using Syrian hamster PrP. By using bank vole PrP we could detect prions in brain samples of reindeer that had tested negative with traditional diagnostic tests. Moreover, we efficiently detected PrP^Sc^ in samples from North American cervids, which have CWD prion strains that might be different from those found in Norway^9^. Thus, the use of bank vole PrP overcomes strain-related effects which are known to influence the efficiency of the RT-QuIC. The capability of bank vole PrP to detect a wide range of prion strains has already been reported^100^ and here we demonstrate for the first time that this substrate enables high efficient detection of multiple CWD strains in different Norwegian CWD affected cervids.

Efficient transmission of TSE infection requires a close similarity between the primary amino acid sequence of the PrP in the donor and in the recipient animal. This allows PrP^Sc^ to interact specifically with and convert the host’s PrP^C^ into the disease-associate isoform and could explain why CWD is so easily transmissible between different cervid species^26,101,102^. Our results showed that the efficiency of the RT-QuIC test was reduced by the use of deer PrP, especially in reindeer, and the sensitivity dropped drastically when using reindeer and elk PrP. Nevertheless, similar observations have been made in the field of human prion diseases: the use of human PrP substrate for RT-QuIC analyses results in lower sensitivity and specificity compared to Syrian hamster or bank vole PrP for detecting PrP^Sc^ in peripheral tissues (e.g. cerebrospinal fluid and olfactory mucosa) of prion diseased patients^103–107^.

PrP sequences of other species, including human and non-cervid ruminants, are dissimilar to that of cervids and this limits the efficiency of interspecies CWD transmission. Nevertheless, the potential risk of interspecies transmission of CWD represents a serious public health concern since humans, cattle and sheep could be exposed to CWD prions through the consumption of prion-infected feed or from contact with a prion-contaminated environment. It is estimated that more than 60% of Americans have eaten deer or elk meat or their derived products^108^ while a large number of cattle, sheep and goats have grazed in CWD contaminated environments. Thankfully, controlled natural exposure studies and targeted surveillance programs currently indicate no cases of natural interspecies transmission of CWD^10,109–113^.

Experimentally, CWD can efficiently be transmitted by intracerebral inoculation in mice, mink, squirrel monkeys, ferrets, sheep and some cattle^10,109,114–118^. But attempts to transmit to transgenic mice overexpressing human prion protein^119^ or to Cynomologus macaques^120^, which are evolutionarily closer to humans, were unsuccessful. This suggests little or no zoonotic potential. The efficiency of CWD transmission to humans has been also evaluated *in vitro* with highly sensitive PMCA and RT-QuIC techniques. Some studies showed that the human PrP can be converted to the pathologic form by CWD prions. It was suggested that the efficiency of this conversion is highly influenced by (i) human PrP polymorphism (recipient), (ii) cervid PrP polymorphism (donor) and (iii) isolates origin (strain)^121–125^. Overall, experiments performed using *in vitro* amplification techniques suggest that the species barrier between cervids and human is not absolute. However, although these techniques mimic *in vitro* the process of prion conversion, they lack many of the biological interactions occurring *in vivo* and the results, regarding the study of the complex phenomenon of the species barrier, should be carefully interpreted. In addition, the species barrier does not only depend on the PrP sequence homology between host and recipient, but also the prion strain. It is therefore conceivable that different CWD strains may have different abilities at crossing the species barrier. Many other factors may play a pivotal role in driving this phenomenon. CWD prions can undergo to processes of selection and adaptation once the interspecies transmission has occurred, with the generation of new prion conformers more prone to propagate in the new host and likely easier to transmit within the species^126,127^. For instance, prions from cattle affected by bovine spongiform encephalopathy (BSE) crossed the species barrier (although with low efficiency) and infected humans, generating a new disease named variant Creutzfeldt-Jakob disease (vCJD)^128^. There is therefore considerable concern that CWD prions could cross the species barrier, adapt to humans and result in new forms of prion disease. The ongoing surveillance has not reported any documented cases of CWD transmission to humans at present. However, the lack of interspecies transmission cannot definitively be ruled out^112,129^.

Our optimized RT-QuIC performed with bank vole PrP could be used as first step screening assay followed by traditional confirmatory TeSeE ELISA, WB or IHC assays to increase the accuracy of CWD detection in affected animals. After a process of validation where many more samples of CWD affected animals and negative controls will be analyzed with this technique, it could be employed as new tool for the diagnosis of CWD either at clinical or preclinical stage of the disease. In addition, considering its elevated analytical sensitivity and rapidity, RT-QuIC might also be exploited for a quick and efficient PrP^Sc^ detection in tissues and biological fluids, such as urine, saliva or feces. These samples are easier to collect than CNS and lymphoid tissues and do not require immobilization or euthanasia of animals. This test could also be used to confirm the absence of infection in animals prior to restocking. Finally, other than monitoring the spreading of CWD prions between cervid species, RT-QuIC with bank vole PrP can be further extended to evaluate the presence of prions in tissues collected from other animals (e.g. sheep, goats, cattle) eventually exposed to contaminated environment.

In conclusion, we provide evidence that RT-QuIC performed with bank vole PrP as reaction substrate is capable of detecting CWD prions, regardless of the cervid species, strains and geographical origin, with good analytical sensitivity and specificity. This rapid and useful technique is, in combination with traditional diagnostic tests, ideal for screening samples containing low concentrations of CWD prions.

## Materials and Methods

### Compliance with Ethical Standards

All animal samples included in this study were provided by the Norwegian surveillance program for CWD in compliance with ethical standards.

### Animals

The following animals from Norway were included in the study: (i) 5 Moose (*Alces alces*) (3 affected by CWD and 2 healthy animals), (ii) 3 red deer (*Cervus elaphus*) (1 affected by CWD and 2 healthy animals) and (iii) 9 reindeer (*Rangifer tarandus tarandus*) (7 affected by CWD and 2 healthy animals). The information concerning the animal’s geographical origin, sex, age and diagnostic status is summarized in Table 2.

### TeSeE^TM^ ELISA and WB tests for CWD diagnosis

All the CWD affected animals were first detected by the Norwegian surveillance program for CWD, using commercially available tests for the detection of PrP^Sc^. Brain tissues and a piece of lymph node were homogenized at 20% (weight/volume) in individual grinding tubes. Rapid test TeSeE^TM^ SAP ELISA (Bio-Rad) was carried out according to the manufacturer’s instruction. Positive ELISA samples were then analyzed with TeSeE^TM^ Western blot (Bio-Rad) for confirmation.

### TeSeE^TM^ Western blot analysis of brain and lymph nodes samples

The homogenates submitted to Western blot were collected from the grinding tubes primarily analyzed by rapid test TeSeE™ ELISA. The WB test was performed, with slight modifications, according to the manufacturer’s instructions. Briefly, PrP^C^ was digested by incubating the homogenates with Proteinase K (20 µl per ml) for 10 min at 37 °C. Electrophoresis was performed using mini-PROTEAN® TGX™ Precast Gels (Bio-Rad) and Power Pac Universal (first 10 min at 60 V followed by approximately 35 min at 120 V). Gels were then electroblotted using semi-dry transfer apparatus (Trans-Blot® Turbo™ Transfer System, Bio-Rad) onto polyvinylidene fluoride (PVDF) membrane (Bio-Rad). The immunoblotting process began by blocking the membrane, to prevent unspecific bindings, with the kit’s block solution for 30 min, then a second 30 min incubation was carried out using monoclonal antibodies SHa31 (AbI from the kit) and an additional monoclonal antibody (P4) at a dilution of 1:1000. Lastly, a 20 min incubation with goat anti-mouse immunoglobulin G (IgG) antibody conjugated with horseradish peroxidase (AbII from the kit) was carried out. The test’s chemiluminescent substrate ECL (Western blotting detecting reagents, Amersham ECL^TM^) was then added and the chemiluminescent signals were visualized using ChemiDoc System (Bio-Rad). The samples were declared positive if characteristic banding patterns of PK-resistant core of PrP^Sc^ were present.

### RT-QuIC recombinant substrates production

Truncated Syrian hamster (90–231), reindeer (94–234; 176D), deer (94–234; 96 G), elk (94–234; 132 M) and bank vole PrP (90–231; 109 M) constructs were purchased from GenScript. The constructs were expressed in Escherichia coli BL21 (DE3) cells (Stratagene). Freshly transformed overnight culture was inoculated into Luria Bertani (LB) medium and 100 μg/mL ampicillin. At 0.8 OD600 expression was induced with isopropyl b-D galactopyranoside (IPTG) to a final concentration of 0.75 mM. Cells were grown in a BioStat-B plus fermentor (Sartorius). The cells were lysed by a homogenizer (PandaPLUS 2000) and the inclusion bodies were suspended in buffer containing 25 mM Tris-HCl, 5 mM EDTA, 0.8% TritonX100, pH 8, and then in bi-distilled water several times. Inclusion bodies containing recombinant proteins were dissolved in 5 volumes of 8 M guanidine hydrochloride (GndHCl), loaded onto pre-equilibrated HiLoad 26/60 Superdex 200-pg column, and eluted in 25 mM Tris–HCl (pH 8), 5 mM ethylenediaminetetraacetic acid (EDTA), and 6 M GndHCl at a flow/rate of 2 mL/min. Proteins refolding was performed by dialysis against refolding buffer (20 mM sodium acetate and 0.005% NaN3 (pH 5.5)) using a Spectrapor membrane. Purified proteins were analyzed by SDS-polyacrylamide gel electrophoresis under reducing conditions and Western blot. Aliquots of the recombinant proteins were stored at −80 °C in 10 mM phosphate buffer (pH 5.8).

### Preparation of the samples for RT-QuIC analyses

Brain tissues were homogenized at 10% (weight/volume) in Bio Rad buffer (from TeSeE^TM^ grinding tubes), serially diluted (from 10^−5^ to 10^−7^) and subjected to RT-QuIC analysis. Two brain tissues of CWD affected white tailed deer (WTd) collected from North America (used for a ring trial for CWD diagnosis in 2006) were homogenized, diluted at 10^−5^ and 10^−6^ and included in the analysis.

### RT-QuIC experimental procedures

Protein substrate solutions were allowed to thaw at room temperature and filtered through a 100 kDa Nanosep centrifugal device (Pall Corporation). Samples were analyzed in triplicate in a black 96-well optical flat bottom plate (ThermoScientific). The final reaction volume was 100 µL and the reagents (Sigma) were concentrated as follow: 150 mM NaCl, 0.002% SDS, 10 mM PBS, 1 mM EDTA, 10 µM ThT and 0.13 mg/ml of recPrP. To avoid contamination, reaction mixes were prepared and loaded (98 µL) onto the microplate in a prion-free laboratory. After the addition of 2 µL of diluted brain homogenates (from 10^−5^ to 10^−7^), the plate was sealed with a sealing film (ThermoScientific) and inserted into a FLUOstar OPTIMA microplate reader (BMG Labtech). The plate was incubated at 55 °C with cycles of 1 min shaking (at 600 rpm, double orbital) and 1 min incubation. Fluorescence readings (480 nm) were taken every 15 min (30 flashes per well at 450 nm). A sample was considered positive if the two highest fluorescence values (AU) of the replicates were greater than 10.000 AU and at least two, out of three replicates, crossed the time threshold that was set for each recombinant substrate. We set the following time thresholds for each PrP: (i) Syrian hamster 48 hours, (ii) bank vole 10 hours, (iii) deer 3.5 hours, (iv) reindeer 3 hours and (v) elk 3 hours. In particular, we have evaluated the time at which the unspecific aggregation of each PrP template occurred in the presence of negative samples (analyzed at least three different times). We have then set this value as time-threshold. Therefore, all samples able to promote PrP aggregation before this time-threshold were considered able to exert a seeding activity while the others were considered unable to promote a seeding activity for each PrP substrate. Data are plotted in graphs showing the time taken for each replicate (black dots) to reach the fluorescence threshold (lag phase).

### Statistical analyses and graphic representation

Statistical analysis (mean and standard error of the mean (S.E.M.)) and graphic representations of our data were performed using the Prism software (v5.0 GraphPad).

## Supplementary information

Supplementary Fig. S1

## Data Availability

All data generated or analyzed during this study are included in this published article.
