# Supplementary material for: Use of different RT-QuIC substrates for detecting CWD prions in the brain of Norwegian cervids
Source: Sci Rep. 2019 Dec 9;9:18595. doi: 10.1038/s41598-019-55078-x (PMC6901582; doi:10.1038/s41598-019-55078-x)
Supplement: Supplementary file 1 — Supplementary Fig. S1 [file 41598_2019_55078_MOESM1_ESM.pdf]

Use of different RT-QuIC substrates for detecting CWD prions in the brain of Norwegian cervids

Edoardo Bistaffa, Tram Thu Vuong, Federico Angelo Cazzaniga, Linh Tran, Giulia Salzano, Giuseppe Legname, Giorgio Giaccone, Sylvie L. Benestad, Fabio Moda

Supplementary Fig. S1

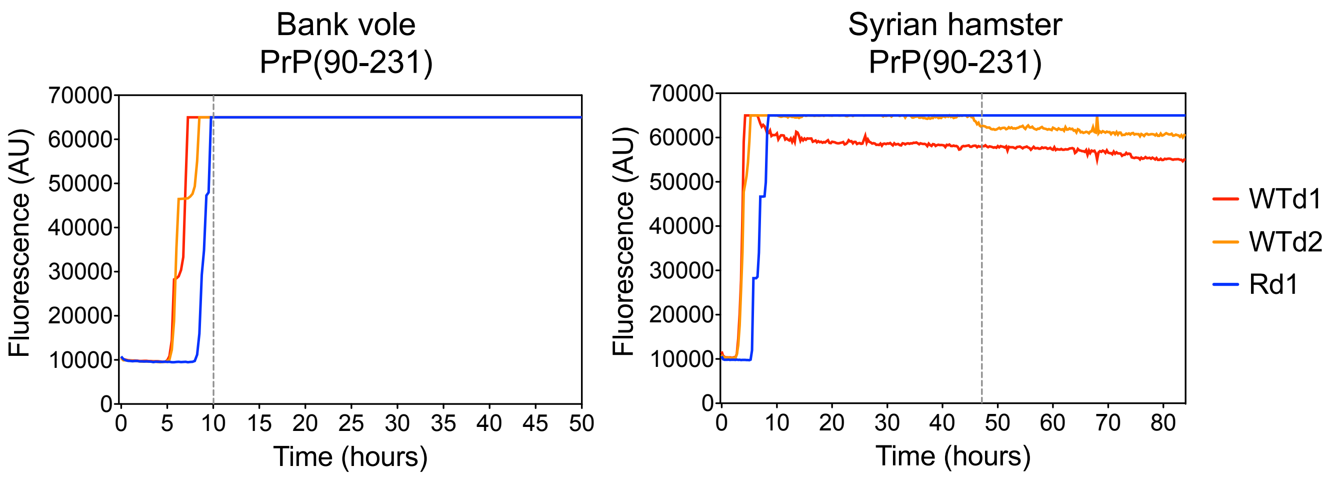

Supplementary Fig. S1. RT-QuIC results of brain from CWD affected Norwegian and North American deer. Brain homogenates were diluted at 10^{-6} (v/v) and analyzed by RT-QuIC using either recombinant bank vole or Syrian hamster truncated PrP. Average ThT fluorescence values were plotted against time. WTd: white tailed deer (North America); Rd: red deer.
